# Dexmedetomidine as an Adjunct to Transversus Abdominis Plane Block in Patients Undergoing Cancer Surgeries: A Systematic Review and Meta-Analysis

**DOI:** 10.3390/life16081217

**Published:** 2026-07-23

**Authors:** Rana Abdulmohsen Alotaibi, Manal Owaid Alharthi, Hazem Ibrahim Shokry, Abdulaziz Fahad Samandar, Wafaa T. Alruwaili, Muzun Hamed Aljohani, Shatha Salem Alhamed, Amal Hussain Al Mutairi, Elaf Abdullah Alruwaili, Rawan Ahmed Alzahrani, Ahmed Tariq Alzahrani, Areej Jarbou Bahrawi, Nawaf Mohammed Alghamdi, Mansour Abdulrahman Alkhulayfi

**Affiliations:** 1 College of Pharmacy, Taif University, Taif 21944, Saudi Arabia; dr.mnol@hotmail.com (M.O.A.); tvcc455@gmail.com (M.A.A.); 2 Anesthesiology and Intensive Care, Ohud Hospital, Minisrty of Heakth, Al Madinah Al Munawwarah 42394, Saudi Arabia; hazemshokry2000@yahoo.com; 3 College of Medicine, Umm Al-Qura University, Makkah 21955, Saudi Arabia; ezoofahad@gmail.com; 4 College of Pharmacy, Northern Border University, Arar 91431, Saudi Arabia; wafaa.t.alruwaili@gmail.com; 5 ‪College of Pharmacy, Taibah University, Al Madinah Al Munawwarah 42353, Saudi Arabia; muzunhalj@gmail.com; 6 Community Pharmacy Outlet Plus, Alhusiba, Dumat al-Jandal 74821, Saudi Arabia; shatha11899@gmail.com; 7 Royal Medical Service, Amman 11855, Jordan; amalalmuatairi@gmail.com; 8 School of Chemistry and Molecular Biosciences (SCMB), The University of Queensland, Brisbane, QLD 4072, Australia; elaf.alrw@gmail.com; 9 ‪College of Pharmacy, Al Baha University, Al Baha 65431, Saudi Arabia; ra.wan.27sa@gmail.com; 10 College of Pharmacy, Prince Sattam Bin Abdulaziz University, Al-Kharj 11942, Saudi Arabia; ahmedtariq1917@gmail.com; 11 College of Medicine, King Abdulaziz University, Jeddah 21589, Saudi Arabia; areejbahrawiwork@gmail.com; 12 College of Pharmacy, King Saud University, Riyadh 11451, Saudi Arabia; 443100273@student.ksu.edu.sa

**Keywords:** transversus abdominis plane block, dexmedetomidine, cancer, surgeries, opioid consumption, postoperative pain

## Abstract

Background: Cancer rates are increasing significantly worldwide, with surgical intervention being the most convenient intervention. Transversus abdominis plane (TAP) block has been proven to enhance postoperative opioid use and its associated complications. Additionally, dexmedetomidine has been reported to provide effective analgesia with relatively minimal respiratory complications. This systematic review and meta-analysis aims to explore the efficacy of combining dexmedetomidine with transversus abdominis plane (TAP) block compared to transversus abdominis plane (TAP) block alone in patients undergoing various cancer surgeries as outcomes may also be influenced by surgical and patient-related factors. Methods: We conducted a comprehensive literature search across PubMed/MEDLINE, Scopus, Web of Science, Cochrane CENTRAL, and Embase to identify randomized controlled trials evaluating the efficacy and safety of dexmedetomidine as an adjunct to transversus abdominis plane (TAP) block in patients undergoing cancer surgery. Data were extracted from the six eligible randomized controlled trials that met the predefined inclusion criteria, and a random-effects meta-analysis was performed using RevMan software Version 5.4. Results: The meta-analysis found that dexmedetomidine as an adjunct to transversus abdominis plane (TAP) block was more effective than transversus abdominis plane (TAP) block alone in reducing the mean arterial pressure and the mean heart rate 2 h postoperatively (MD −6.46, 95% CI [−8.63; −4.30], *p* < 0.0001) and (MD −5.44, 95% CI [−7.67; −3.20], *p* < 0.0001). The duration until the first rescue analgesia was significantly longer in the TAP–DEX group (MD 3.75, 95% CI [3.08; 4.43], *p* < 0.0001). The overall patient satisfaction with postoperative pain management demonstrated statistically significant improvement compared to the TAP group (RR 1.31, 95% CI [1.13; 1.53], *p* = 0.0005). visual analog scale (VAS) pain scores, which are used to assess pain intensity, were significantly lower at rest and during movement 2 h postoperatively in the TAP-DEX group, although some complex patients may have difficulty using visual analog scale (VAS) pain scores accurately. Therefore, the Numeric Rating Scale (NRS) may be considered as an alternative tool. Conclusions: Compared to transversus abdominis plane (TAP) block alone, the dexmedetomidine and transversus abdominis plane (TAP) block arm showed a statistically significant difference in time to first rescue analgesia, patient overall satisfaction, nausea, and vomiting. However, no statistical significance difference was found across the remaining outcomes.

## 1. Introduction

In 2020, the number of new reported cancer cases rose dramatically to 19.3 million around the globe, making it a leading cause of mortality and morbidity worldwide [1,2]. Cancer resection intervention is the gold-standard treatment option for cancer management protocols [3]. Additionally, every year, approximately 100,000 cancer resection surgeries are conducted in the United States alone [3]. These surgeries contribute greatly to relieving cancer symptoms, improving overall recovery outcomes and the patient’s quality of life [3]. Nonetheless, cancer resection surgeries carry the risk of many complications such as postoperative pain and gastrointestinal disturbances like nausea and vomiting [4,5,6]. These complications often result in psychological and economic burdens for patients due to prolonged treatment periods and poorer outcomes [7,8]. Appropriate postoperative analgesia is crucial to limit postoperative pain that may lead to extended hospital stay and slower recovery [9].

The transversus abdominis plane block is a promising regional anesthesia approach that targets the nerves originating from T6 to L1 [10,11]. This regional anesthesia aims to block the nerves supplying the anterior abdominal wall [10,11]. For effective anesthesia, anesthetic agents are injected between the internal oblique and transversus abdominis muscles, specifically into the fascial plane [11,12,13]. Respiratory depression is one of the most serious complications associated with opioid consumption perioperatively [14,15]. Transversus abdominis plane (TAP) block provides a protective mechanism for the respiratory system by reducing opioid needs postoperatively [16,17]. However, transversus abdominis plane (TAP) block may not be enough to provide complete anesthesia for different types of patients across various surgical contexts [18]. Moreover, the efficacy outcomes after transversus abdominis plane (TAP) block depend on many subjective factors, including the patient’s anatomy and the skills of the healthcare provider performing the technique [19,20]. Furthermore, transversus abdominis plane (TAP) block alone may be incompatible in terms of other postoperative complications such as sedation levels and nausea [21].

The analgesic and sedative properties of dexmedetomidine, a highly selective alpha-2 adrenergic agonist, have been widely proven [22,23]. In addition to its effective analgesia and sedation properties, dexmedetomidine was found to have minimal effects on respiratory depression, making it a promising choice for complex surgeries [24,25,26]. Dexmedetomidine targets the central nervous system (CNS) through reducing the release of norepinephrine into the CNS [27]. This mechanism modulates neurotransmitter release and contributes to the drug’s analgesic and sedative effects, especially when adding local anesthetics such as bupivacaine [28,29]. Therefore, dexmedetomidine has proven significant efficacy in maintaining hemodynamic stability [30,31].

The integration of transversus abdominis plane (TAP) block and dexmedetomidine has been reported to better enhance patients’ recovery outcomes than using transversus abdominis plane (TAP) block alone in different surgical contexts [32]. Given the sedative and analgesic properties of dexmedetomidine, besides its efficacy in decreasing opioid consumption and safety by lowering respiratory depression, adding it to transversus abdominis plane (TAP) block may provide lower VAS scores. Additionally, it could provide a more stable postoperative state and improve patient satisfaction [33]. This could ultimately be translated into shorter hospital stays and lower healthcare costs due to decreased complications associated with inadequate pain control [32,33].

This systematic review and meta-analysis aims to explore the efficacy of dexmedetomidine as an adjunct to transversus abdominis plane block on mean heart rate, mean arterial pressure, VAS at rest, VAS at movement, time to 1st rescue analgesia, sedation score, nausea, vomiting, and patient satisfaction among patients undergoing different cancer surgeries in comparison with using transversus abdominis plane block alone.

## 2. Materials and Methods

### 2.1. Protocol and Registration

A preset protocol for this systematic review and meta-analysis was developed and published in PROSPERO with the registration number of [CRD420261292180]. We conducted this systematic review and meta-analysis according to the Preferred Reporting Items for Systematic Reviews and Meta-Analyses (PRISMA) checklist for systematic reviews [34] and under the clear guidance of the Cochrane Handbook of Systematic Reviews and Meta-analysis of Interventions (version 5.1.0) [35].

### 2.2. Eligibility Criteria

#### Inclusion and Exclusion Criteria

The inclusion and exclusion criteria for this systematic review were carefully designed to ensure the selection of high-quality studies that aligned with the research objectives and provided reliable evidence. Eligible studies were required to fulfill the following Population, Intervention, Comparison, Outcomes, and Study Design (PICOS) criteria:

i. Population: Patients undergoing cancer surgeries.
ii. Intervention: Dexmedetomidine as an adjunct to transversus abdominis plane (TAP) block.
iii. Control: Transversus abdominis plane (TAP) block alone.
iv. Outcomes: Studies reporting at least one of the following outcomes of interest:
  - Mean heart rate;
  - Mean arterial pressure;
  - Visual analog scale (VAS) pain score at rest;
  - Visual analog scale (VAS) pain score during movement;
  - Time to first rescue analgesia;
  - Sedation score, assessed using validated sedation scales such as the Ramsay Sedation Scale (RSS) or Richmond Agitation–Sedation Scale (RASS), when sufficient extractable data were available;
  - Postoperative nausea and vomiting;
  - Patient satisfaction.
v. Study design: Randomized controlled trials.

##### Exclusion Criteria

The following studies were excluded:

i. Study designs other than randomized controlled trials, including observational studies, case reports, case series, reviews, and editorials;
ii. Studies involving patients undergoing surgeries other than cancer surgeries;
iii. Studies that did not directly assess any of the predefined outcomes of interest;
iv. Studies published in languages other than English.

### 2.3. Information Sources and Search Strategy

A comprehensive electronic literature search was conducted in PubMed/MEDLINE, Scopus, Web of Science, Cochrane Central Register of Controlled Trials (CENTRAL), and Embase from database inception to 29 January 2025. Clinical trial registry databases were also searched to identify completed or published randomized controlled trials. The search strategy was developed using a combination of controlled vocabulary terms, including Medical Subject Headings (MeSHs) where applicable and free-text keywords related to dexmedetomidine, transversus abdominis plane block, cancer surgery, and postoperative analgesia. Boolean operators “AND” and “OR” were used to combine search concepts. No restrictions were applied regarding publication year; however, only studies published in English were included.

The PubMed/MEDLINE search strategy was as follows:

(“Dexmedetomidine” [Mesh] OR dexmedetomidine OR “alpha-2 agonist” OR “alpha 2 agonist”) AND (“Transversus Abdominis Plane Block” [Mesh] OR “transversus abdominis plane block” OR “TAP block” OR “abdominal plane block”) AND (“Neoplasms” [Mesh] OR cancer OR neoplasm OR malignancy OR tumor OR tumour OR oncologic OR oncology) AND (“Surgical Procedures, Operative” [Mesh] OR surgery OR surgical OR postoperative OR perioperative) AND (“Pain, Postoperative” [Mesh] OR analgesia OR “postoperative pain” OR “opioid consumption” OR “rescue analgesia”).

The Scopus search strategy was as follows:

TITLE-ABS-KEY (dexmedetomidine OR “alpha-2 agonist” OR “alpha 2 agonist”) AND TITLE-ABS-KEY (“transversus abdominis plane block” OR “TAP block” OR “abdominal plane block”) AND TITLE-ABS-KEY (cancer OR neoplasm OR malignancy OR tumor OR tumour OR oncologic OR oncology) AND TITLE-ABS-KEY (surgery OR surgical OR postoperative OR perioperative) AND TITLE-ABS-KEY (analgesia OR “postoperative pain” OR “opioid consumption” OR “rescue analgesia”).

The Web of Science search strategy was as follows:

TS = (dexmedetomidine OR “alpha-2 agonist” OR “alpha 2 agonist”) AND TS = (“transversus abdominis plane block” OR “TAP block” OR “abdominal plane block”) AND TS = (cancer OR neoplasm OR malignancy OR tumor OR tumour OR oncologic OR oncology) AND TS = (surgery OR surgical OR postoperative OR perioperative) AND TS = (analgesia OR “postoperative pain” OR “opioid consumption” OR “rescue analgesia”).

The Cochrane CENTRAL search strategy was as follows:

(dexmedetomidine OR “alpha-2 agonist” OR “alpha 2 agonist”) AND (“transversus abdominis plane block” OR “TAP block” OR “abdominal plane block”) AND (cancer OR neoplasm OR malignancy OR tumor OR tumour OR oncologic OR oncology) AND (surgery OR surgical OR postoperative OR perioperative) AND (analgesia OR “postoperative pain” OR “opioid consumption” OR “rescue analgesia”).

The Embase search strategy was as follows:

(‘dexmedetomidine’/exp OR dexmedetomidine OR ‘alpha 2 agonist’ OR ‘alpha-2 agonist’) AND (‘transversus abdominis plane block’/exp OR ‘transversus abdominis plane block’ OR ‘TAP block’ OR ‘abdominal plane block’) AND (‘cancer’/exp OR cancer OR neoplasm OR malignancy OR tumor OR tumour OR oncologic OR oncology) AND (‘surgery’/exp OR surgery OR surgical OR postoperative’OR perioperative) AND (‘postoperative pain’/exp OR analgesia OR ‘postoperative pain’ OR ‘opioid consumption’ OR ‘rescue analgesia’).

The complete electronic search strategy for all databases is provided in Supplementary Table S1 to ensure reproducibility and compliance with PRISMA recommendations.

### 2.4. Study Selection and Data Extraction

All records identified through the electronic database searches were imported into EndNote 21 software for reference management and duplicate removal [36]. The remaining records were then uploaded to the Rayyan online platform to facilitate independent screening [37]. Two reviewers independently screened the titles and abstracts of all retrieved records according to the predefined eligibility criteria. Studies that clearly did not meet the inclusion criteria were excluded at this stage.

Full-text reports of potentially eligible studies were retrieved and assessed independently by two reviewers. Any disagreements regarding study eligibility were resolved through discussion, and, when necessary, by consultation with a third reviewer. Reasons for exclusion at the full-text screening stage were documented and summarized in the PRISMA 2020 flow diagram. The complete list of full-text-excluded articles, including the reason for exclusion for each article, is provided in Supplementary Table S2.

Data from the included studies were extracted into a standardized Google sheet [38]. Extracted information included the first author’s name, year of publication, country, study design, sample size, surgical procedure, intervention and comparator details, baseline patient characteristics, and outcomes of interest.

### 2.5. Assessment of Risk of Bias

The methodological quality of the included studies was assessed using the revised Cochrane risk-of-bias tool for randomized trials (RoB 2) [39]. This tool evaluates potential sources of bias across five domains: bias arising from the randomization process, bias due to deviations from the intended interventions, bias due to missing outcome data, bias in measurement of the outcome, and bias in selection of the reported result. Each domain was judged as having low risk of bias, some concerns, or high risk of bias according to the RoB 2 guidance. Two reviewers independently assessed the risk of bias for each included study. Any disagreements were resolved through discussion, and, when necessary, by consultation with a third reviewer. After domain-level assessment, an overall risk-of-bias judgment was assigned for each study.

### 2.6. Statistical Analysis

We conducted the meta-analysis using the meta package in R software, version 4.3.3 [40]. Dichotomous outcomes were pooled as risk ratios (RRs), while continuous outcomes were pooled as mean differences (MDs), each with corresponding 95% confidence intervals.

For multi-arm randomized controlled trials that included more than one dexmedetomidine dose group and a single shared control group, eligible dexmedetomidine intervention arms were combined into one pooled TAP-DEX group before inclusion in the meta-analysis. For dichotomous outcomes, the number of events and total participants were summed across the eligible dexmedetomidine arms. For continuous outcomes, combined means and standard deviations were calculated according to the formulas recommended in the Cochrane Handbook. This approach was used to avoid double-counting the shared control group and to minimize the risk of unit-of-analysis errors or inappropriate study weighting.

We employed a random-effects model because it provides a more conservative estimate of the pooled effect when clinical or methodological heterogeneity is expected. Heterogeneity was initially assessed through visual inspection of forest plots and was further evaluated using the Chi-square test and I^2^ statistic. A probability value of *p* ≤ 0.10 for the Chi-square test was considered indicative of statistically significant heterogeneity. Sensitivity analyses were conducted by excluding one study at a time for each outcome to assess the robustness of the pooled estimates. When outcome data were available only in graphical form, values were extracted using WebPlotDigitizer version 4.7. When necessary, the Meta Convert Tool was used to estimate means and standard deviations from medians and ranges [41].

## 3. Results

### 3.1. Study Selection

The systematic literature search identified 218 potentially relevant records across four electronic databases: PubMed (*n* = 14), Cochrane CENTRAL (*n* = 38), Web of Science (*n* = 18), and Scopus (*n* = 148). No additional records were identified from registers. After removal of 38 duplicate records, 180 records were screened by title and abstract. Of these, 151 records were excluded because they did not meet the predefined eligibility criteria.

**Figure 1:**
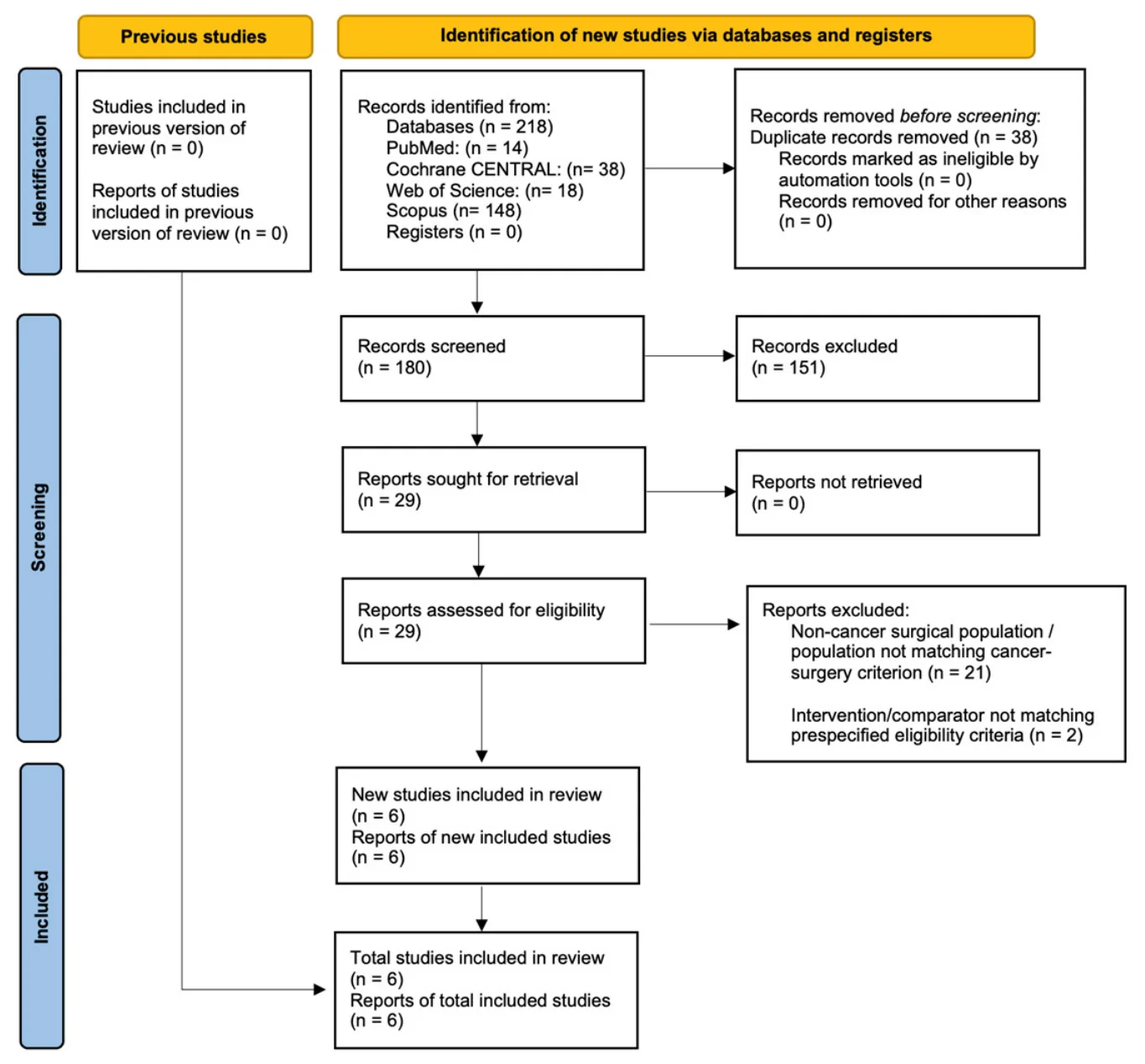
PRISMA 2020 flow diagram showing the identification, screening, eligibility assessment, and inclusion of studies in the systematic review and meta-analysis.

A total of 29 reports were sought for retrieval and assessed for eligibility. No reports were unavailable for retrieval. Following full-text assessment, 23 reports were excluded: 21 because the study population did not match the cancer-surgery criterion and 2 because the intervention or comparator did not match the prespecified eligibility criteria. Finally, six studies were included in both the qualitative and quantitative synthesis. The study selection process is presented in the revised PRISMA 2020 flow diagram (Figure 1).

### 3.2. Study Characteristics

After literature screening, our meta-analysis included six publications that met the eligibility criteria, all following a prospective randomized controlled trial design conducted from 2020 to 2024, including 416 patients. Four of the studies were conducted in China while two were conducted in Egypt. Follow-up periods and participant baseline characteristics varied between studies (Table 1). Information on the demographics and features of the included studies is provided in detail in (Table 2).

### 3.3. Quality Assessment

**Figure 2:**
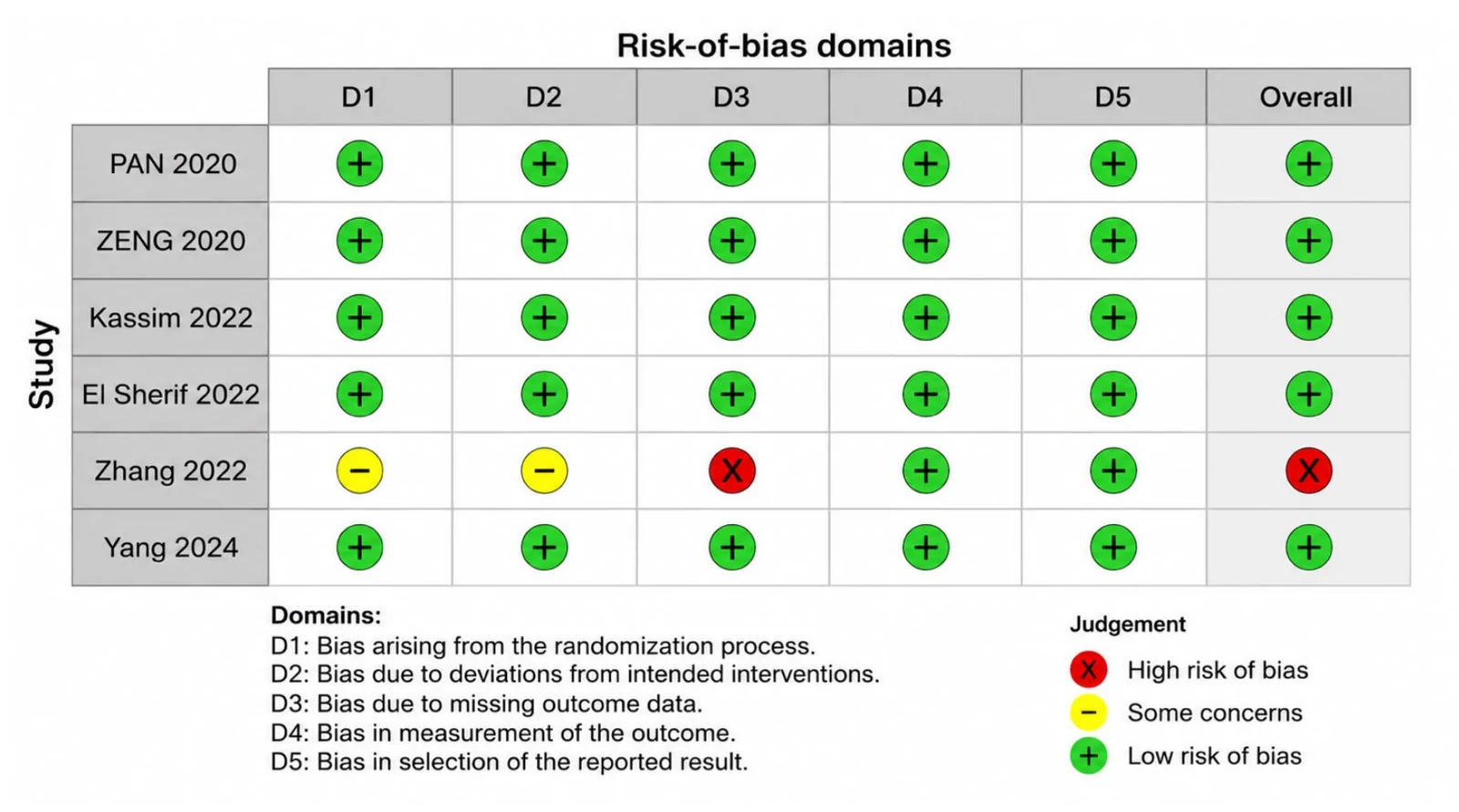
Quality assessment of the included studies using the Cochrane Risk of Bias 2 (RoB 2) assessment tool [39]. Data are derived from the included studies by PAN et al. 2020 [42], ZENG et al. 2020 [43], El Sherif et al. 2022 [44], Kassim et al. 2022 [45], Zhang et al. 2022 [47], Yang et al. 2024 [46].

The quality of the included studies was assessed using the risk-of-bias assessment tool (ROB-2) for randomized controlled trials [39]. Five of the included studies were judged as low risk [42,43,44,45,46] while one study exhibited high risk [47]. Detailed information about the studies’ quality assessment is shown in Figure 2.

### 3.4. Outcomes

#### 3.4.1. Mean Arterial Pressure

**Figure 3:**
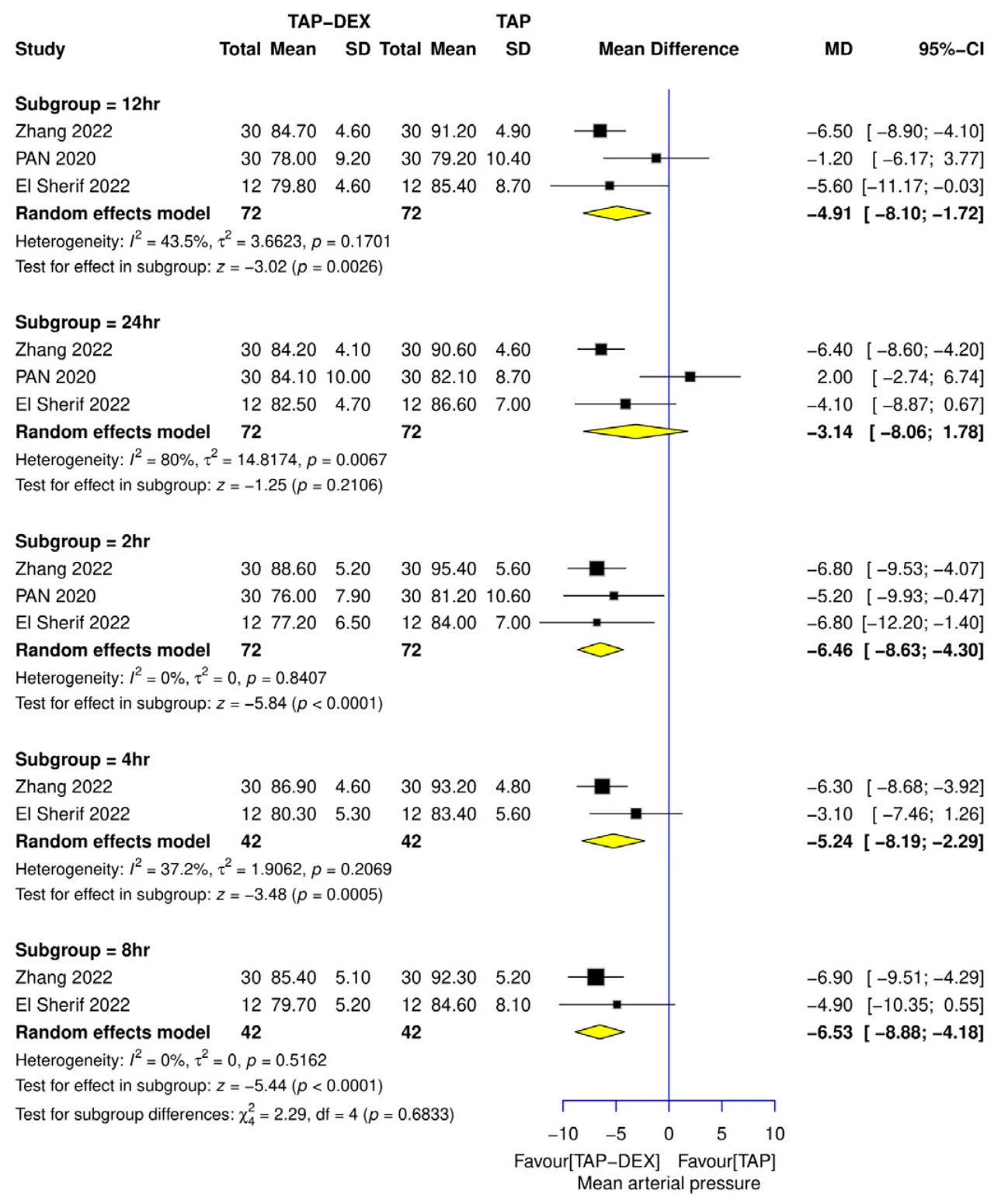
Postoperative mean arterial pressure. Data are derived from the included studies by PAN et al. [42], El Sherif et al. [44] Zhang et al. [47].

Three included studies (PAN 2020, Zhang 2022, and El Sherif 2022) [42,44,47], comprising a total of 144 patients, reported postoperative mean arterial pressure (MAP). Stratified analyses according to the postoperative assessment time demonstrated significantly lower MAP values in the TAP–DEX group at 2, 4, 8, and 12 h compared with the TAP-only group (2 h: MD = −6.46, 95% CI [−8.63, −4.30], *p* < 0.0001; 4 h: MD = −5.24, 95% CI [−8.19, −2.29], *p* = 0.0005; 8 h: MD = −6.53, 95% CI [−8.88, −4.18], *P* < 0.0001; 12 h: MD = −4.91, 95% CI [−8.10, −1.72], *p* = 0.0026). In contrast, no statistically significant difference was observed at 24 h (MD = −3.14, 95% CI [−8.06, 1.78], *p* = 0.2106) (Figure 3).

These findings suggest that the addition of dexmedetomidine to the TAP block may provide improved early postoperative hemodynamic stability, whereas this effect was not sustained at 24 h.

**Figure 4:**
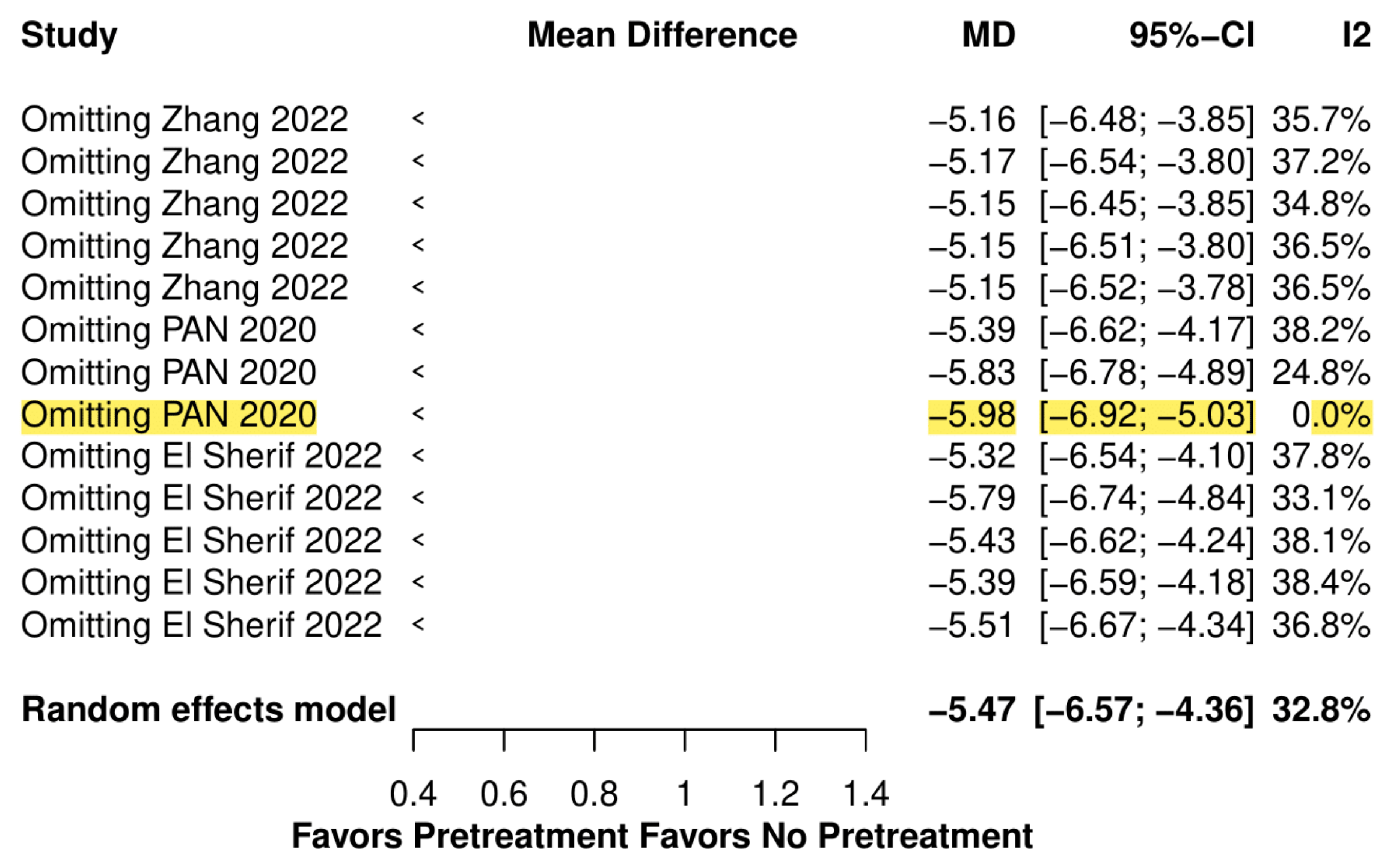
Sensitivity analysis of postoperative mean arterial pressure. Data are derived from the included studies by PAN et al. [42], El Sherif et al. [44] Zhang et al. [47].

Heterogeneity was observed only in the 24 h subgroup (χ^2^ = 14.8, *p* = 0.0067; I^2^ = 80%). Sensitivity analysis, performed by sequentially excluding one study at a time (Figure 4), demonstrated that the observed heterogeneity was primarily attributable to the PAN 2020 study [42].

**Table 1:** Characteristics of the included studies.

Study ID	PAN 2020 [42]	ZENG 2020 [43]	El Sherif 2022 [44]	Kassim 2022 [45]	Zhang 2022 [47]	Yang 2024 [46]
Study Design	Prospective, randomized, double-blind clinical study	Prospective, randomized, double-blinded, controlled clinical trial	Prospective randomized, double-blind clinical controlled study	Prospective, randomized and single-center research design	Prospective, randomized, double-blind clinical study	Randomized, double-blind, controlled trial
Registry	http://www.chictr.org.cn (accessed on 5 September 2025) (chictr-IOR-17014122)	(CHICTR; www.chictr.org.cn; registration no. Chictr1900020995)	NCT03328299	NCT04318158	(Approval No. 2018030012)	NA
Country	China	China	Egypt	Egypt	China	China
Surgery	Laparoscopic colectomy	Laparotomy for gynecologic malignancies	Lower abdominal cancer surgery	Radical cystectomy	Colon cancer surgery	Radical colorectal cancer surgery
Intervention (I)	20 mL 0.375% ropivacaine plus 2 mL of Dex (0.5 µg/kg)	0.5 µg/kg dexmedetomidine or 1 µg/kg or 2 µg/kg with 50 mL of 0.3% ropivacaine	20 mL 0.5% bupivacaine + dexmedetomidine 1 μg/kg	TAP block: 20 mL 0.25% bupivacaine + dexmedetomidine 1 μg/kg	Dexmedetomidine 0.5, 1.0, or 1.5 μg/kg + 0.25% ropivacaine (20 mL per side)	0.5% ropivacaine + 1 μg/kg dexmedetomidine (amounting to 20 mL)
Control (C)	20 mL 0.375% ropivacaine + 2 mL normal saline	Transversus abdominis plane (TAP) block with 50 mL of 0.3% ropivacaine	Bilateral single-injection TAP block with 20 mL 0.5% bupivacaine	Single-shot ultrasound-guided TAP block with 20 mL 0.25% bupivacaine + 2 mL normal saline	Same volume of normal saline	0.5% ropivacaine 20 mL was injected into each transversus abdominis plane
Sample size	31	25 each	12	20	30 each	90
General Anesthesia Induction	Midazolam, sufentanil, propofol, and cisatracurium	Midazolam, sufentanil, propofol, and rocuronium	Propofol, fentanyl, lidocaine, and rocuronium	Propofol, fentanyl, and atracurium	Sufentanil, propofol, and cisatracurium	Sufentanil, midazolam, cisatracurium, and etomidate
General Anesthesia Maintenance	Propofol and remifentanil	Propofol, remifentanil and sevoflurane	Isoflurane in a 50% oxygen/air mixture	Isoflurane and fentanyl infusion	Propofol, remifentanil, and cisatracurium	Remifentanil and propofol
Muscle Relaxation	Cisatracurium	Cisatracurium	Rocuronium	Atracurium	Cisatracurium	Cisatracurium

**Table 2:** Baseline characteristics of included studies.

Study ID	Study Arms	n	Age (y), Mean (SD)	Gender (M:F)	Body Mass Index (BMI) (kg/m^2^), Mean (SD)	Weight (kg), Mean (SD)	American Society of Anesthesiologists (ASA) (I/II)	Operation Time (minutes), Mean (SD)	Hemorrhage (mL), Mean (SD)
Zhang 2022 [47]	Dexmedetomidine (low dose) 0.2–0.3	30	46.01 (8.21)	13:17	24.47 (2.32)	63.12 (7.02)	20/10	161.2 (32.7)	160.26 (25.18)
	Dexmedetomidine (moderate dose) 0.3–0.5	30	45.67 (8.35)	12:18	25.02 (2.08)	63.26 (6.86)	23/7	165.1 (34.2)	162.63 (25.31)
	Dexmedetomidine (high dose) 0.5–1	30	46.57 (9.04)	11:19	24.63 (2.05)	63.53 (7.29)	19/11	163.52 (32.3)	161.34 (24.68)
	Control	30	45.94 (8.24)	12:18	23.89 (2.14)	62.85 (6.28)	17/13	165.1 (34.2)	159.63 (25.31)
Kassim 2022 [45]	Dexmedetomidine	20	61.50 (6.82)	19/1	25.30 (2.94)	NA	14/6	6.40 (0.82) *	NA
	Control	20	60.40 (6.85)	17/3	24.75 (2.31)	NA	16/4	6.80 (0.77) *	NA
Yang 2024 [46]	Dexmedetomidine	85	62.16 (6.68)	27/58	23.16 (3.33)	62.00 (10.29)	NA	4.27 (1.16) *	244.23 (161.41)
	Control	84	63.61 (7.08)	34/50	23.00 (3.62)	59.65 (10.22)	NA	4.05 (1.09) *	216.19 (138.04)
ZENG 2020 [43]	0.5 μg/kg dexmedetomidine	25	50.16 (6.16)	NA	20.92 (1.81)	NA	13/12	180.56 (34.23)	340.20 (79.81)
	1 μg/kg dexmedetomidine	25	48.64 (7.17)	NA	20.91 (1.63)	NA	14/11	175.40 (34.70)	338.20 (83.24)
	2 μg/kg dexmedetomidine	26	48.73 (9.10)	NA	20.99 (1.65)	NA	12:14	185.08 (32.57)	326.80 (85.58)
	Control	25	49.28 (8.23)	NA	21.04 (1.74)	NA	11:14	181.92 (33.02)	317.00 (107.94)
PAN 2020 [42]	Dexmedetomidine	30	60.2 (13.5)	17/13	23.6 (5.1)	71.3 (14.4)	NA	178.8 (48.6)	192 (94)
	Control	30	61.5 (12.3)	16/4	23.3 (4.8)	69.2 (15.6)	NA	172.6 (52.5)	187 (69)
El Sherif 2022 [44]	Dexmedetomidine	12	53.08 (9.6)	5 (7)	28.47 (4.81)	76.92 (8.43)	9 (3)	173.00 (11.01)	NA
	Control	12	52.58 (8.54)	7 (5)	27.09 (3.08)	76.33 (7.98)	8 (4)	173.92 (17.89)	NA

*: Numbers are presented in hours not minutes.

#### 3.4.2. Mean Heart Rate

**Figure 5:**
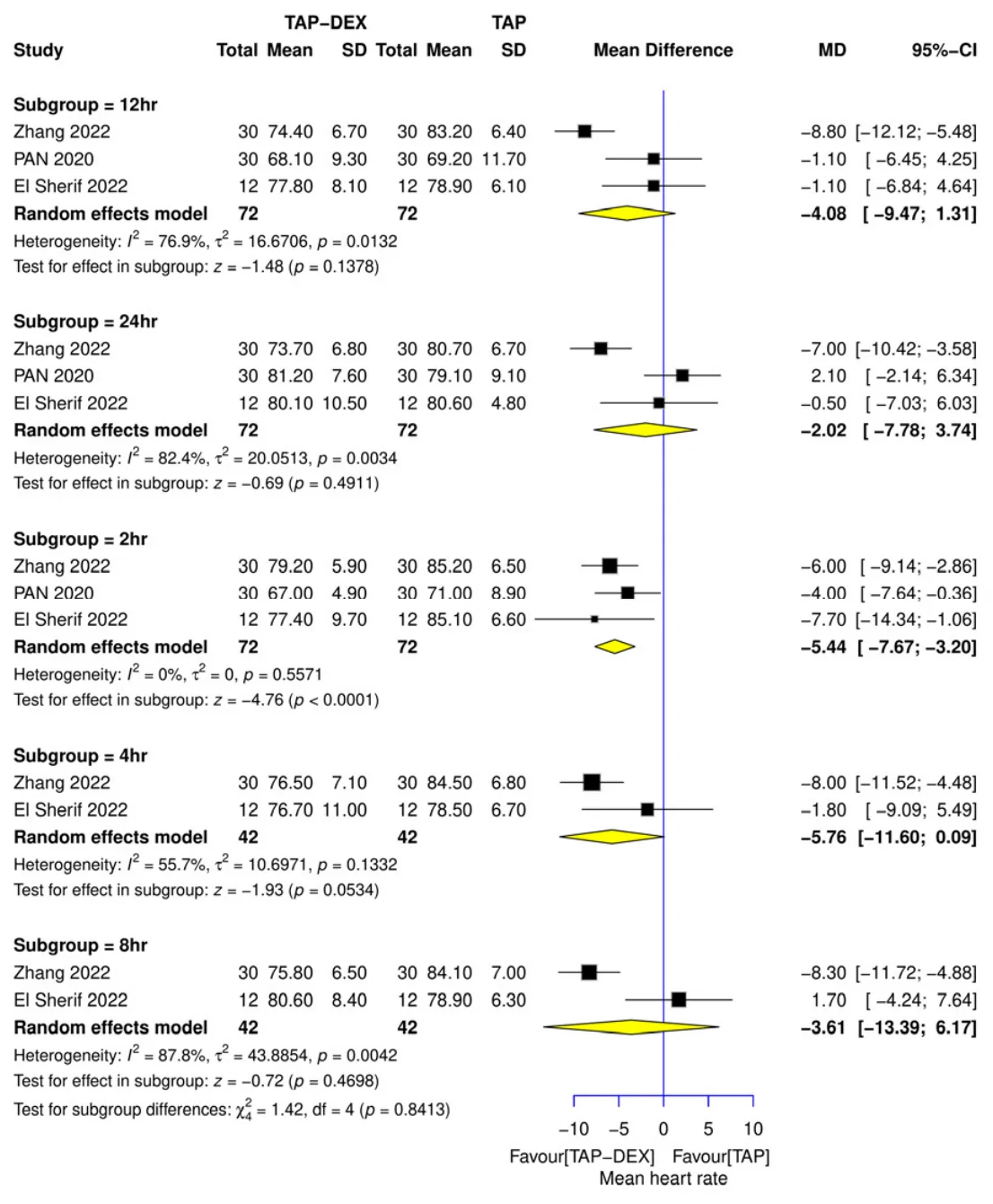
Postoperative mean heart rate. Data are derived from the included studies by PAN et al. [42], El Sherif et al. [44] Zhang et al. [47].

Three included studies (PAN 2020, Zhang 2022, and El Sherif 2022) [42,44,47], comprising a total of 144 patients, reported postoperative heart rate (HR). Stratified analyses according to postoperative assessment time demonstrated a significantly lower heart rate in the TAP–DEX group only at 2 h compared with the TAP-only group (MD = −5.44, 95% CI [−7.67, −3.20], *p* < 0.0001). No statistically significant differences were observed at 4, 8, 12, or 24 h (4 h: MD = −5.76, 95% CI [−11.60, 0.09], *p* = 0.0534; 8 h: MD = −3.61, 95% CI [−13.39, 6.17], *P* = 0.4698; 12 h: MD = −4.08, 95% CI [−9.47, 1.31], *p* = 0.1378; 24 h: MD = −2.02, 95% CI [−7.78, 3.74], *p* = 0.4911) (Figure 5).

These findings indicate that the addition of dexmedetomidine to the TAP block may provide a transient reduction in postoperative heart rate during the early recovery period, whereas this effect was not maintained at later postoperative time points. Significant heterogeneity was identified across several subgroup analyses and could not be resolved through sensitivity analysis (Figure S1).

#### 3.4.3. Time to First Rescue Analgesia

Four included studies (PAN 2020, ZENG 2020, El Sherif 2022, and Kassim 2022) [42,43,44,45], comprising 174 patients, reported the time to first rescue analgesia. The pooled analysis demonstrated a significantly longer time to first rescue analgesia in the TAP–DEX group compared with the TAP-only group (MD = 3.75, 95% CI [3.08, 4.43], *p* < 0.0001), with no evidence of heterogeneity (Chi-square = 0.06, *p* = 0.421, I^2^ = 0%) (Figure S2).

These findings suggest that dexmedetomidine may prolong the duration of postoperative analgesia and delay the need for additional rescue analgesics.

#### 3.4.4. Patient Overall Satisfaction

Three included studies (PAN 2020, ZENG 2020, and Kassim 2022) [42,43,45], including 150 patients, reported overall patient satisfaction. The pooled analysis demonstrated significantly greater overall patient satisfaction in the TAP–DEX group than in the TAP-only group (RR = 1.31, 95% CI [1.13, 1.53], *p* = 0.0005), with no observed heterogeneity (Chi-square = 0, *p* = 0.712, I^2^ = 0%) (Figure S3).

This finding indicates that the improved postoperative analgesic profile associated with dexmedetomidine may contribute to higher patient satisfaction.

#### 3.4.5. Sedation Score

Sedation score was prespecified as an outcome of interest. However, quantitative synthesis was not performed because sedation outcomes were not reported consistently across the included studies or were not available in a sufficiently extractable format using comparable sedation scales and time points. Therefore, sedation score was not included in the pooled meta-analysis. This limitation should be considered when interpreting the overall safety and postoperative recovery profile of dexmedetomidine as an adjunct to transversus abdominis plane block.

#### 3.4.6. Visual Analog Pain Scores (VAS) at Rest

Four included studies (PAN 2020, El Sherif 2022, Kassim 2022, and Yang 2024) [42,44,45,46], involving 293 patients, reported postoperative VAS pain scores at rest. A significant reduction in pain scores was observed in the TAP–DEX group at 6 h postoperatively (MD = −0.91, 95% CI [−1.77, −0.06], *p* = 0.0363). No statistically significant differences were identified at 12 h (MD = −0.33, 95% CI [−1.07, 0.40], *p* = 0.371) or 24 h (MD = −0.27, 95% CI [−0.62, 0.09], *p* = 0.1398) (Figure S4).

These results suggest that the analgesic benefit of dexmedetomidine is most evident during the early postoperative period rather than at later time points. Significant heterogeneity was detected across the subgroup analyses and could not be resolved through sensitivity analysis (Figure S5).

#### 3.4.7. Visual Analog Pain Scores (VAS) at Movement

Three included studies (PAN 2020, El Sherif 2022, and Kassim 2022) [42,44,45], comprising 124 patients, reported postoperative VAS pain scores during movement. The TAP–DEX group demonstrated significantly lower pain scores at 6 h (MD = −1.30, 95% CI [−2.43, −0.18], *p* = 0.0234). No statistically significant differences were observed at 12 h (MD = −0.39, 95% CI [−1.42, 0.65], *p* = 0.466) or 24 h (MD = −0.10, 95% CI [−0.44, 0.24], *p* = 0.561) (Figure S6).

These findings indicate that dexmedetomidine may improve pain control during movement primarily in the early postoperative period. Significant heterogeneity was detected across subgroup analyses and could not be resolved through sensitivity analysis (Figure S7).

#### 3.4.8. Postoperative Nausea

Five included studies (PAN 2020, ZENG 2020, Zhang 2022, El Sherif 2022, and Kassim) 2022 [42,43,44,45,47], representing 234 patients, reported postoperative nausea. The pooled analysis demonstrated a significantly lower incidence of postoperative nausea in the TAP–DEX group than in the TAP-only group (RR = 0.37, 95% CI [0.20, 0.69], *p* = 0.0018), with no evidence of heterogeneity (Chi-square = 0, *p* = 0.693, I^2^ = 0%) (Figure S8).

This reduction may reflect improved postoperative analgesia and reduced opioid requirements in patients receiving dexmedetomidine.

#### 3.4.9. Postoperative Vomiting

Five included studies (PAN 2020, ZENG 2020, Zhang 2022, El Sherif 2022, and Kassim) 2022 [42,43,44,45,47], involving 234 patients, reported postoperative vomiting. The pooled analysis showed a significantly lower incidence of postoperative vomiting in the TAP–DEX group compared with the TAP-only group (RR = 0.25, 95% CI [0.11, 0.54], *p* = 0.0005), without significant heterogeneity (Chi-square = 0, *p* = 0.771, I^2^ = 0%) (Figure S9).

These findings suggest that the addition of dexmedetomidine may contribute to improved postoperative recovery by reducing emesis.

## 4. Discussion

Effective postoperative pain management is essential for enhancing recovery, facilitating early mobilization, reducing opioid-related adverse effects, and improving patient satisfaction following cancer surgery. Transversus abdominis plane (TAP) block has become an important component of multimodal analgesia because it provides effective somatic analgesia of the anterior abdominal wall while reducing perioperative opioid requirements [48,49]. However, the analgesic efficacy of TAP block may vary according to several factors, including patient characteristics, anatomical variability, surgical procedure, and block technique, which may limit the consistency of postoperative pain control [50]. Dexmedetomidine, a highly selective α2-adrenergic receptor agonist, possesses analgesic, sedative, and sympatholytic properties with minimal respiratory depression [51,52]. As an adjuvant to local anesthetics, dexmedetomidine has the potential to prolong the duration of regional anesthesia, improve analgesic efficacy, and enhance postoperative recovery [42,43,44,45,46,47]. Therefore, combining dexmedetomidine with TAP block represents a promising strategy for optimizing perioperative analgesia in patients undergoing cancer surgery.

This systematic review and meta-analysis demonstrate that adding dexmedetomidine to the transversus abdominis plane (TAP) block provides several clinically relevant benefits for patients undergoing cancer surgery. Overall, the pooled evidence suggests that the TAP–DEX technique improves postoperative analgesia, prolongs the duration of pain relief, enhances patient satisfaction, and reduces the incidence of postoperative nausea and vomiting compared with TAP block alone [42,43,44,45,46,47]. In addition, favorable short-term effects on hemodynamic parameters were observed, indicating that dexmedetomidine may contribute to improved perioperative stability [42,43,44,45,46,47]. Collectively, these findings support the use of dexmedetomidine as an effective adjuvant to TAP block within multimodal analgesia protocols for cancer surgery, although the magnitude and persistence of these benefits may vary according to patient characteristics, surgical procedures, and dexmedetomidine dosing strategies.

Our pooled analysis demonstrated that the addition of dexmedetomidine to the transversus abdominis plane (TAP) block was associated with significantly lower mean arterial pressure during the first 12 postoperative hours, suggesting improved short-term hemodynamic stability. This effect is biologically plausible because dexmedetomidine exerts central sympatholytic activity through selective α2-adrenergic receptor agonism, thereby reducing sympathetic outflow and attenuating the physiological stress response to surgery [51,52]. Such hemodynamic stability may be particularly beneficial in patients undergoing major cancer surgery, especially those with underlying cardiovascular comorbidities or an increased perioperative cardiovascular risk. However, the absence of a significant difference at 24 h suggests that these hemodynamic effects are primarily confined to the early postoperative period. Although heterogeneity was minimal for this outcome except at the 24 h time point, variations in dexmedetomidine dosage, local anesthetic regimens, postoperative assessment times, and the diversity of surgical procedures among the included studies may have contributed to the observed variability [42,43,44,45,46,47].

Similarly, the addition of dexmedetomidine to the transversus abdominis plane (TAP) block was associated with a significant reduction in heart rate during the early postoperative period. This finding is consistent with the pharmacological profile of dexmedetomidine, which reduces sympathetic nervous system activity through central α2-adrenergic receptor stimulation, thereby attenuating the cardiovascular response to surgical stress [51,52]. A lower postoperative heart rate may reflect improved analgesia and reduced sympathetic activation rather than clinically significant bradycardia, particularly when accompanied by stable mean arterial pressure. However, this effect was not sustained beyond the early postoperative period, which is likely attributable to the transient sympatholytic effects of dexmedetomidine following single-dose administration. It is also important to note that substantial heterogeneity was observed for this outcome. Variations in dexmedetomidine dose, patient characteristics, perioperative anesthetic management, and the diversity of cancer surgical procedures among the included studies may have contributed to this variability, highlighting the need for larger, standardized randomized controlled trials to better define the cardiovascular effects of dexmedetomidine as an adjunct to TAP block [42,43,44,45,46,47].

One of the most clinically relevant findings of this meta-analysis was the significantly prolonged time to first rescue analgesia observed in patients receiving dexmedetomidine as an adjunct to the transversus abdominis plane (TAP) block [42,43,44,45,46,47]. This finding suggests that dexmedetomidine enhances both the quality and duration of postoperative analgesia, thereby reducing the early need for supplemental opioid analgesics. Prolonged analgesia is particularly advantageous in patients undergoing cancer surgery, as effective pain control facilitates early mobilization, improves patient comfort, and may contribute to enhanced recovery after surgery (ERAS) protocols. Furthermore, reducing postoperative opioid requirements may decrease the incidence of opioid-related adverse effects, including nausea, vomiting, excessive sedation, ileus, and delayed recovery [48,49]. Although the included studies reported consistent benefits for this outcome with minimal statistical heterogeneity, differences in dexmedetomidine dosage, local anesthetic concentration, and the type and extent of cancer surgery may still influence the magnitude of analgesic benefit. Therefore, future randomized controlled trials comparing different dexmedetomidine dosing strategies across specific surgical populations are warranted to establish the optimal adjuvant regimen.

### 4.1. Clinical Implications

The findings of this systematic review and meta-analysis suggest that dexmedetomidine is a promising adjuvant to the transversus abdominis plane (TAP) block for patients undergoing cancer surgery. The addition of dexmedetomidine was associated with prolonged postoperative analgesia, improved patient satisfaction, reduced postoperative nausea and vomiting, and favorable short-term hemodynamic effects without compromising the overall safety profile reported in the included studies [42,43,44,45,46,47]. These benefits support the incorporation of dexmedetomidine into multimodal analgesia protocols, particularly within enhanced recovery after surgery (ERAS) pathways, where minimizing opioid consumption and promoting early postoperative recovery are important objectives [48,49].

Nevertheless, these findings should be interpreted with appropriate caution. The included studies varied in dexmedetomidine dose, local anesthetic regimen, surgical procedures, and postoperative outcome assessment, all of which may influence the observed treatment effects. Consequently, although the current evidence supports the clinical utility of dexmedetomidine as an adjunct to TAP block, further large-scale, high-quality randomized controlled trials are needed to determine the optimal dosing strategy, evaluate long-term safety, and identify the patient populations most likely to benefit from this approach.

### 4.2. Limitations

Several limitations should be considered when interpreting the findings of this systematic review and meta-analysis. First, the number of included studies was limited, and the overall sample size was relatively small, which may reduce the precision and generalizability of the pooled estimates. Second, the included trials varied in several clinically important aspects, including cancer surgery type, dexmedetomidine dose, local anesthetic regimen, TAP block technique, anesthesia protocol, and postoperative assessment time points. These differences may have contributed to the heterogeneity observed in some outcomes, particularly heart rate and VAS pain scores. Third, although multi-arm trials were managed statistically to avoid double-counting of control groups, variations in dexmedetomidine dosing across studies limited our ability to determine the optimal dose. Fourth, sedation score was prespecified as an outcome of interest but could not be quantitatively synthesized because the included studies did not report sedation outcomes consistently or in a sufficiently extractable format using comparable scales and time points. Fifth, most outcomes were assessed only during the early postoperative period; therefore, the long-term analgesic efficacy and safety of dexmedetomidine as an adjunct to TAP block remain uncertain. Finally, publication bias could not be formally assessed because fewer than ten studies were available for each outcome, which limits confidence in detecting small-study effects. Future large-scale, well-designed randomized controlled trials with standardized dosing, surgical subgroups, outcome definitions, and longer follow-up are needed to confirm these findings.

## 5. Conclusions

In conclusion, this systematic review and meta-analysis suggests that adding dexmedetomidine to transversus abdominis plane (TAP) block can improve the outcomes of cancer surgery patients by enhancing postoperative recovery and patient satisfaction. However, future research is needed to explore the optimal dosing of dexmedetomidine alongside transversus abdominis plane (TAP) block as well as to explore the long-term outcomes of this combination. Furthermore, more studies are required to investigate the optimal techniques and administration routes for this intervention, aiming to increase their advantages and reduce the potential adverse events.

## Data Availability

All data analyzed during this study are included in this published article and its Supplementary Information files.

## Supplementary Materials

The following supporting information can be downloaded at: https://www.mdpi.com/article/10.3390/life16081217/s1.
