# Supplementary material for: Dexmedetomidine as an Adjunct to Transversus Abdominis Plane Block in Patients Undergoing Cancer Surgeries: A Systematic Review and Meta-Analysis
Source: Life (Basel). 2026 Jul 23;16(8):1217. doi: 10.3390/life16081217 (PMC13514275; doi:10.3390/life16081217)
Supplement: Supplementary file 1 [file life-16-01217-s001.zip › supplementary figures.pdf]

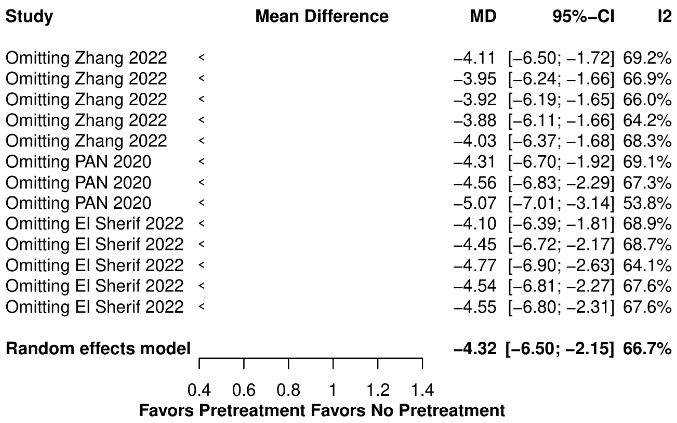

Figure S1. Sensitivity analysis of mean heart rate

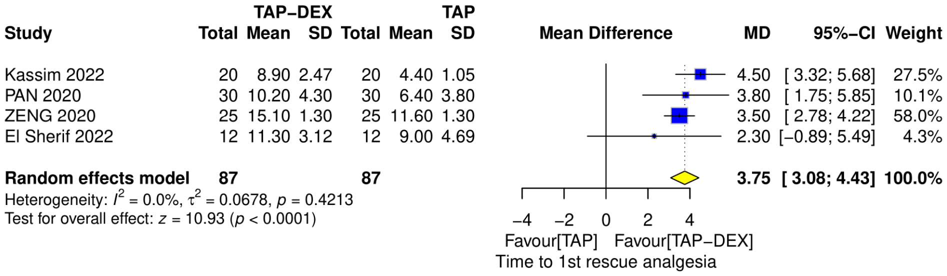

Figure S2. Mean difference in time to first rescue analgesia

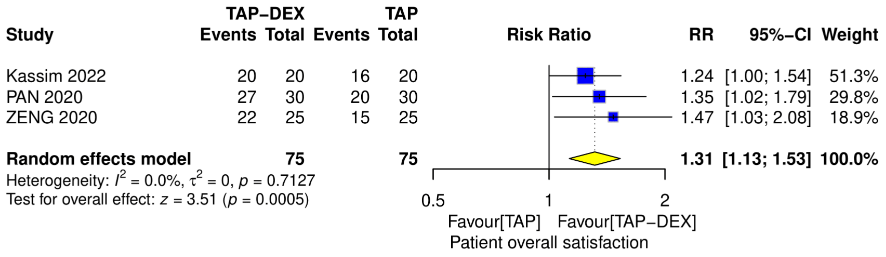

Figure S3. Patient overall satisfaction

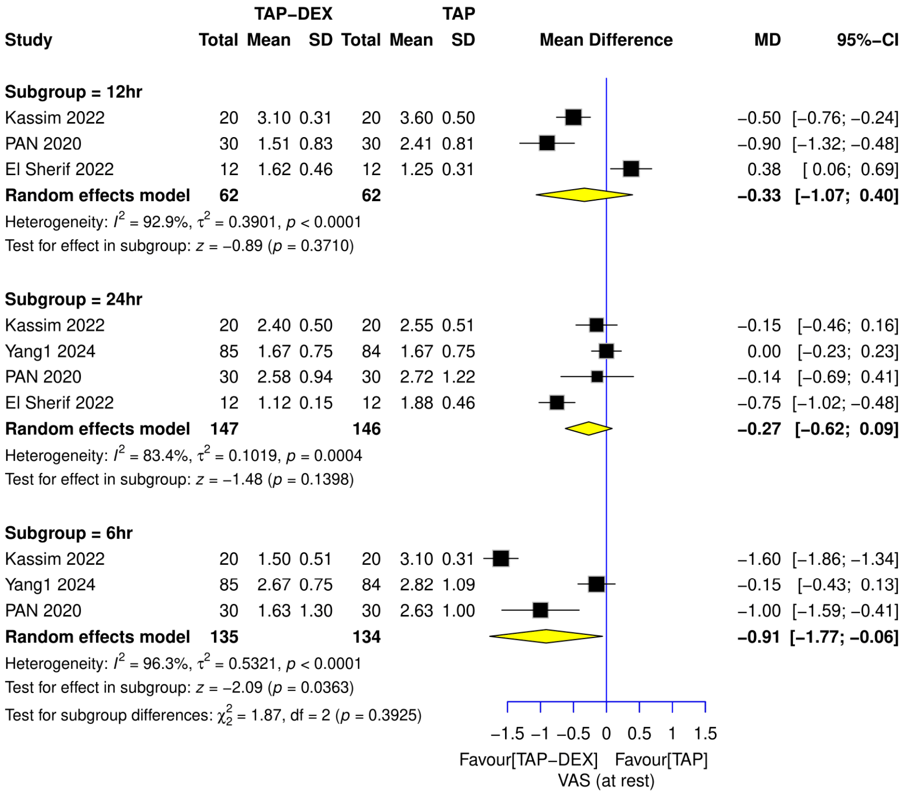

Figure S4. Postoperative VAS at rest

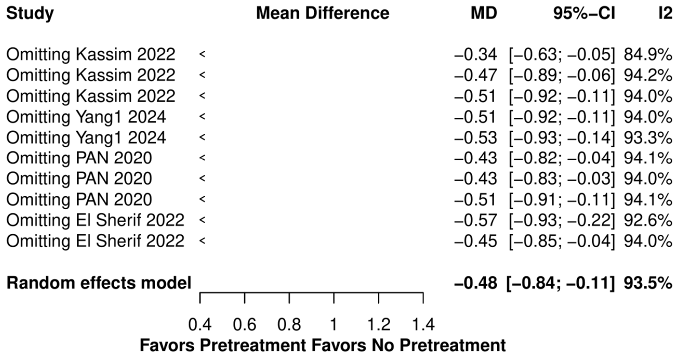

Figure S5. Sensitivity analysis of Postoperative VAS at rest

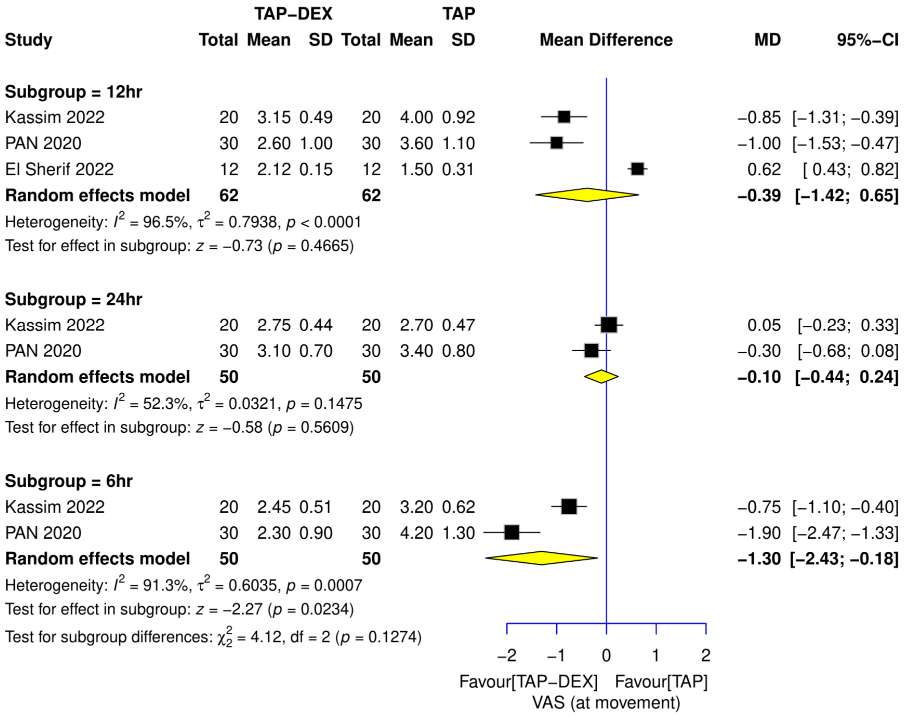

Figure S6. Postoperative VAS at movment

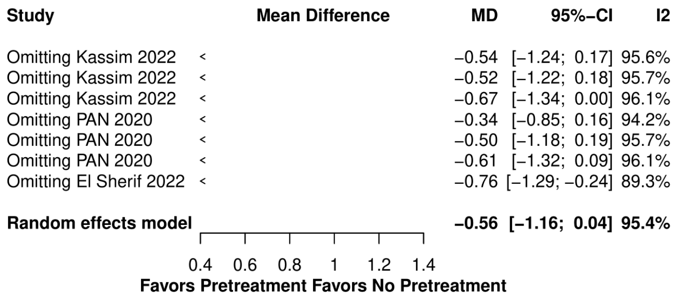

Figure S7. Sensitivity analysis of Postoperative VAS at movement

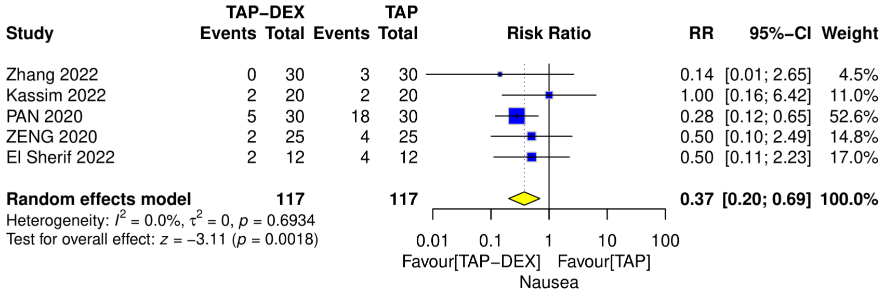

Figure S8. Nausea

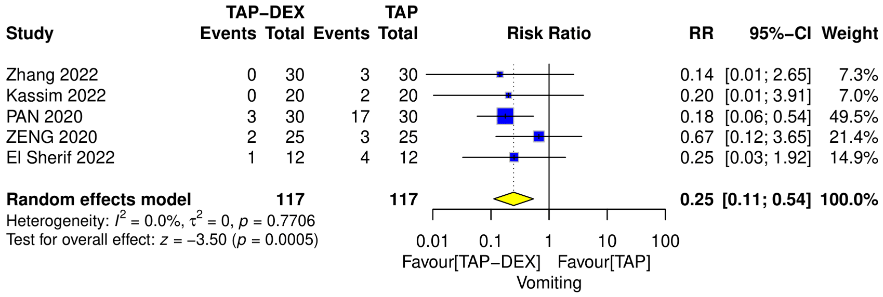

Figure S9. Vomiting
