# Supplementary material for: Dexmedetomidine as an Adjunct to Transversus Abdominis Plane Block in Patients Undergoing Cancer Surgeries: A Systematic Review and Meta-Analysis
Source: Life (Basel). 2026 Jul 23;16(8):1217. doi: 10.3390/life16081217 (PMC13514275; doi:10.3390/life16081217)
Supplement: Supplementary file 1 [file life-16-01217-s001.zip › Supplementary_Table_S1_Search_Strategy.pdf]

Supplementary Table S1. Complete electronic search strategy used for each database

Search date: January 29, 2025. Searches were conducted from database inception. The search combined controlled vocabulary terms, where available, with free-text terms using Boolean operators. Only English-language randomized controlled trials were eligible for inclusion.

Database/source	Electronic search strategy
**PubMed/MEDLINE**	("Dexmedetomidine"[Mesh] OR dexmedetomidine OR "alpha-2 agonist" OR "alpha 2 agonist") AND ("Transversus Abdominis Plane Block"[Mesh] OR "transversus abdominis plane block" OR "TAP block" OR "abdominal plane block") AND ("Neoplasms"[Mesh] OR cancer OR neoplasm OR malignancy OR tumor OR tumour OR oncologic OR oncology) AND ("Surgical Procedures, Operative"[Mesh] OR surgery OR surgical OR postoperative OR perioperative) AND ("Pain, Postoperative"[Mesh] OR analgesia OR "postoperative pain" OR "opioid consumption" OR "rescue analgesia").
**Scopus**	TITLE-ABS-KEY (dexmedetomidine OR "alpha-2 agonist" OR "alpha 2 agonist") AND TITLE-ABS-KEY ("transversus abdominis plane block" OR "TAP block" OR "abdominal plane block") AND TITLE-ABS-KEY (cancer OR neoplasm OR malignancy OR tumor OR tumour OR oncologic OR oncology) AND TITLE-ABS-KEY (surgery OR surgical OR postoperative OR perioperative) AND TITLE-ABS-KEY (analgesia OR "postoperative pain" OR "opioid consumption" OR "rescue analgesia").
**Web of Science**	TS=(dexmedetomidine OR "alpha-2 agonist" OR "alpha 2 agonist") AND TS=("transversus abdominis plane block" OR "TAP block" OR "abdominal plane block") AND TS=(cancer OR neoplasm OR malignancy OR tumor OR tumour OR oncologic OR oncology) AND TS=(surgery OR surgical OR postoperative OR perioperative) AND TS=(analgesia OR "postoperative pain" OR "opioid consumption" OR "rescue analgesia").
**Cochrane CENTRAL**	(dexmedetomidine OR "alpha-2 agonist" OR "alpha 2 agonist") AND ("transversus abdominis plane block" OR "TAP block" OR "abdominal plane block") AND (cancer OR neoplasm OR malignancy OR tumor OR tumour OR oncologic OR oncology) AND (surgery OR surgical OR postoperative OR perioperative) AND (analgesia OR "postoperative pain" OR "opioid consumption" OR "rescue analgesia").
**Embase**	('dexmedetomidine'/exp OR dexmedetomidine OR 'alpha 2 agonist' OR 'alpha-2 agonist') AND ('transversus abdominis plane block'/exp OR 'transversus abdominis plane block' OR 'TAP block' OR 'abdominal plane block') AND ('cancer'/exp OR cancer OR neoplasm OR malignancy OR tumor OR tumour OR oncologic OR oncology) AND ('surgery'/exp OR surgery OR surgical OR postoperative OR perioperative) AND ('postoperative pain'/exp OR analgesia OR 'postoperative pain' OR 'opioid consumption' OR 'rescue analgesia').
**Clinical trial registries**	(dexmedetomidine OR "alpha-2 agonist" OR "alpha 2 agonist") AND ("transversus abdominis plane block" OR "TAP block") AND (cancer OR malignancy OR neoplasm OR oncology) AND (surgery OR postoperative OR perioperative).

Abbreviations: CENTRAL, Cochrane Central Register of Controlled Trials; MeSH, Medical Subject Headings; TAP, transversus abdominis plane.
