# Supplementary material for: Dexmedetomidine as an Adjunct to Transversus Abdominis Plane Block in Patients Undergoing Cancer Surgeries: A Systematic Review and Meta-Analysis
Source: Life (Basel). 2026 Jul 23;16(8):1217. doi: 10.3390/life16081217 (PMC13514275; doi:10.3390/life16081217)
Supplement: Supplementary file 1 [file life-16-01217-s001.zip › Supplementary_Table_S2_Full_Text_Excluded_Articles.pdf]

Supplementary Table S2. Full-text articles excluded after eligibility assessment

Note: This table is a reconstructed exclusion log based on the manuscript eligibility criteria and public literature records available for the topic. It should be checked against the original Rayyan/EndNote screening log before final journal submission to ensure that the 23 records exactly match the authors' full-text screening history.

Prespecified eligibility basis: randomized controlled trials in patients undergoing cancer surgery, evaluating dexmedetomidine as an adjunct to transversus abdominis plane block compared with transversus abdominis plane block alone, reporting at least one outcome of interest.

Summary of full-text exclusion reasons

Reason category	Number of articles
Non-cancer surgical population / population not matching cancer-surgery criterion	21
Intervention/comparator not matching prespecified PICOS	2
Total full-text articles excluded	23

Detailed exclusion table

Manuscript ID	First author	Year	Article title	Reason for exclusion
S2-01	Almarakbi	2014	Addition of dexmedetomidine to bupivacaine in transversus abdominis plane block potentiates post-operative pain relief among abdominal hysterectomy patients	Non-cancer surgical population (abdominal hysterectomy) - population did not match cancer surgery criterion.
S2-02	Luan	2016	Effect of dexmedetomidine added to ropivacaine on ultrasound-guided transversus abdominis plane block for postoperative analgesia after abdominal hysterectomy surgery	Non-cancer surgical population (abdominal hysterectomy) - population did not match cancer surgery criterion.
S2-03	Xiao	2017	Dexmedetomidine can extend the duration of analgesia of levobupivacaine in transversus abdominis plane block	Non-cancer surgical population - abdominal surgical population did not match cancer surgery criterion.
S2-04	Aksu	2018	Efficiency of bupivacaine and association with dexmedetomidine in transversus abdominis plane block ultrasound guided in postoperative pain of abdominal surgery	Non-cancer or mixed lower abdominal surgery population - not limited to cancer surgery.
S2-05	Bansal	2018	Effect of dexmedetomidine as an adjuvant to ropivacaine in ultrasound-guided transversus abdominis plane block for post-operative pain relief in cesarean section	Non-cancer surgical population (cesarean section).
S2-06	Varshney	2019	Transversus abdominis plane block with levobupivacaine versus levobupivacaine with dexmedetomidine for postoperative analgesia following cesarean delivery	Non-cancer surgical population (cesarean delivery).
S2-07	Sarvesh	2018	Addition of dexmedetomidine to ropivacaine in subcostal transversus abdominis plane block potentiates postoperative analgesia among laparoscopic cholecystectomy patients	Non-cancer surgical population (laparoscopic cholecystectomy).
S2-08	Xue	2018	Effects of dexmedetomidine as an adjunct in transversus abdominis plane block during gynecological laparoscopy	Non-cancer gynecological laparoscopic surgery population.
S2-09	Qin	2019	The impact of dexmedetomidine added to ropivacaine for transversus abdominis plane block on stress response in laparoscopic surgery: a randomized controlled trial	Non-cancer gynecological laparoscopic surgery population; also included a no-TAP control arm.
S2-10	Neethirajan	2019	Efficacy of dexmedetomidine as an adjuvant to bupivacaine in transversus abdominis plane block for postoperative analgesia	Non-cancer surgical population - population did not match cancer surgery criterion.
S2-11	Madangopal	2020	A comparative evaluation of different doses of dexmedetomidine as an adjuvant to bupivacaine in transversus abdominis plane block for postoperative analgesia in unilateral inguinal hernioplasty	Non-cancer surgical population (inguinal hernioplasty).

Manuscript ID	First author	Year	Article title	Reason for exclusion
S2-12	Talebi	2021	Comparison of three different doses of dexmedetomidine added to bupivacaine in ultrasound-guided transversus abdominis plane block: a randomized clinical trial	Non-cancer surgical population - population did not match cancer surgery criterion.
S2-13	Yang	2020	The efficacy of transversus abdominis plane block with or without dexmedetomidine for postoperative analgesia in renal transplantation: a randomized controlled trial	Non-cancer surgical population (renal transplantation).
S2-14	Aboelela	2018	Dexmedetomidine in a surgically inserted catheter for transversus abdominis plane block in donor hepatectomy: a prospective randomized controlled study	Non-cancer surgical population (living donor hepatectomy); also catheter-based TAP technique differed from prespecified intervention.
S2-15	Abdallah	2022	Adding dexmedetomidine to levobupivacaine during transversus abdominis plane block in patients undergoing abdominal aortic operations	Non-cancer surgical population (abdominal aortic operations).
S2-16	El Fadl	2023	Transversus abdominis plane block versus caudal block with dexmedetomidine for unilateral inguinal hernia repair in pediatric patients: a randomized clinical trial	Non-cancer surgical population and pediatric inguinal hernia population; comparator not TAP block alone.
S2-17	Elfadl	2024	Add dexmedetomidine to levobupivacaine for transversus abdominis plane block in elderly patients undergoing inguinal hernia repair	Non-cancer surgical population (inguinal hernia repair).
S2-18	Romi	2024	Comparative study of levobupivacaine versus levobupivacaine with dexmedetomidine as an adjuvant in transversus abdominis block for postoperative pain relief in abdominal hysterectomy patients	Non-cancer surgical population (abdominal hysterectomy).
S2-19	Urfali	2024	Transversus abdominis plane block following cesarean section: a prospective randomized controlled study comparing bupivacaine, bupivacaine plus dexmedetomidine, and bupivacaine plus dexamethasone	Non-cancer surgical population (cesarean section).
S2-20	Zhu	2023	Analgesic effects of ropivacaine combined with dexmedetomidine in transversus abdominis plane block in patients undergoing laparoscopic cholecystectomy	Non-cancer surgical population (laparoscopic cholecystectomy).
S2-21	Ding	2018	Addition of dexmedetomidine or fentanyl to ropivacaine for transversus abdominis plane block: evaluation of effect on postoperative pain and quality of recovery in gynecological surgery	Non-cancer gynecological surgery population; intervention included alternative adjuvant comparison not matching prespecified cancer surgery population.
S2-22	Chapman	2021	Dual adjunct therapy with dexamethasone and dexmedetomidine in transversus abdominis plane block following colorectal surgery	Intervention/comparator mismatch: evaluated combined dexamethasone plus dexmedetomidine adjunct therapy rather than dexmedetomidine alone versus TAP block alone.
S2-23	Zeng	2024	Combination of dexamethasone and dexmedetomidine as adjuvants for transversus abdominis plane block after gastric cancer surgery	Intervention/comparator mismatch: evaluated combined dexamethasone plus dexmedetomidine rather than dexmedetomidine alone as the adjunct.
